# Supplementary material for: End Sequence Analysis Toolkit (ESAT) expands the extractable information from single-cell RNA-seq data
Source: Genome Res. 2016 Oct;26(10):1397–410. doi: 10.1101/gr.207902.116 (PMC5052061; doi:10.1101/gr.207902.116)
Supplement: Supplemental Material [file supp_gr.207902.116_Supp_Materials.tar.gz › ESAT-esat_v0.1/doc/html/classumms_1_1core_1_1coordinatesystem_1_1_transcript_in_genomic_space.html]

ESAT: umms.core.coordinatesystem.TranscriptInGenomicSpace Class Reference


ESAT


- Main Page
- Related Pages
- Packages
- Classes
- Files

- Class List
- Class Index
- Class Hierarchy
- Class Members

All Classes Namespaces Files Functions Variables Enumerator Pages

- umms
- core
- coordinatesystem
- TranscriptInGenomicSpace

Classes |
Public Member Functions |
Static Public Member Functions |
Static Public Attributes |
List of all members

umms.core.coordinatesystem.TranscriptInGenomicSpace Class Reference

Inheritance diagram for umms.core.coordinatesystem.TranscriptInGenomicSpace:

[legend]

Collaboration diagram for umms.core.coordinatesystem.TranscriptInGenomicSpace:

[legend]

|  |  |
| --- | --- |
| Public Member Functions | |
|  | TranscriptInGenomicSpace (Map< String, Integer > chromosomeSizes) |
|  | |
|  | TranscriptInGenomicSpace (String chromosomeSizes) |
|  | |
|  | TranscriptInGenomicSpace (String chromosomeSizes, String maskedRegionFile) |
|  | |
|  | TranscriptInGenomicSpace (Map< String, Integer > chromosomeSizes, String maskedRegionFile) |
|  | |
|  | TranscriptInGenomicSpace (String chromosomeSizes, String maskedRegionFile, double pctMaskedAllowed) |
|  | |
| void | setPercentMaskedAllowed (double pct) |
|  | |
| Collection<?extends Window > | getOverlappingRegion (String chr, int start, int end) |
|  | |
| Iterator< Window > | getWindowIterator (int windowSize, String chr, int start, int end, int overlap) |
|  | |
| Iterator< Window > | getWindowIterator (int windowSize, String chr, int start, int end, int overlap, boolean backward) |
|  | |
| Iterator<?extends Window > | getWindowIterator (Annotation window, int windowSize, int overlap) |
|  | |
| Iterator<?extends Window > | getWindowIterator (Annotation window, int windowSize, int overlap, boolean considerStrand) |
|  | |
| Iterator< Window > | getWindowIterator (String chr, int windowSize, int overlap) |
|  | |
| Iterator< Window > | getWindowIterator (int windowSize, int overlap) |
|  | |
| boolean | isValidWindow (Annotation window) |
|  | |
| boolean | containsDataFor (String chromosome) |
|  | |
| long | getLength () |
|  | |
| long | getLength (String chr) |
|  | |
| long | getUnmaskedLength (String chr) |
|  | |
| Annotation | permuteAnnotation (Annotation a) |
|  | |
| boolean | hasChromosome (String chr) |
|  | |
| Annotation | permuteAnnotation (Annotation a, Annotation bounds) |
|  | |
| final Collection< String > | getReferenceNames () |
|  | |
| final Collection<?extends   Annotation > | getReferenceAnnotations () |
|  | |
| Annotation | getEntireChromosome (String chrName) |
|  | |
| Annotation | getReferenceAnnotation (String name) |
|  | |
| Iterator< Window > | getWindowIterator (Collection< Gene > baseGenes, int windowSize, int overlap) |
|  | |
| Collection<?extends Window > | getFragment (String chr, int start, int end) |
|  | |
| Collection<?extends Window > | getFragment (Annotation annotation) |
|  | |
| int | getSize (Annotation region) |
|  | |
| Collection< String > | getChromosomeNames () |
|  | |

|  |  |
| --- | --- |
| Static Public Member Functions | |
| static void | setSeed (long seed) |
|  | |

|  |  |
| --- | --- |
| Static Public Attributes | |
| static final int | PERMUTATION\_ATTEMPTS = 10 |
|  | |

## Detailed Description

Author
:   skadri Class representing a genome with defined linear chromosomes. Masking behavior can be controlled through setting masked regions and percent masked allowed parameters, which will filter out windows that overlap masked regions.

## Constructor & Destructor Documentation

|  |  |  |  |  |  |
| --- | --- | --- | --- | --- | --- |
| umms.core.coordinatesystem.TranscriptInGenomicSpace.TranscriptInGenomicSpace | ( | Map< String, Integer > | *chromosomeSizes* | ) |  |

Parameters
:   |  |  |
    | --- | --- |
    | chromosomeSizes |  |

|  |  |  |  |  |  |
| --- | --- | --- | --- | --- | --- |
| umms.core.coordinatesystem.TranscriptInGenomicSpace.TranscriptInGenomicSpace | ( | String | *chromosomeSizes* | ) |  |

Chromosome name and accompanying sizes

Parameters
:   |  |  |
    | --- | --- |
    | chromosomeSizes |  |

|  |  |  |  |
| --- | --- | --- | --- |
| umms.core.coordinatesystem.TranscriptInGenomicSpace.TranscriptInGenomicSpace | ( | String | *chromosomeSizes*, |
|  |  | String | *maskedRegionFile* |
|  | ) |  |  |

|  |  |  |  |
| --- | --- | --- | --- |
| umms.core.coordinatesystem.TranscriptInGenomicSpace.TranscriptInGenomicSpace | ( | Map< String, Integer > | *chromosomeSizes*, |
|  |  | String | *maskedRegionFile* |
|  | ) |  |  |

|  |  |  |  |
| --- | --- | --- | --- |
| umms.core.coordinatesystem.TranscriptInGenomicSpace.TranscriptInGenomicSpace | ( | String | *chromosomeSizes*, |
|  |  | String | *maskedRegionFile*, |
|  |  | double | *pctMaskedAllowed* |
|  | ) |  |  |

Here is the call graph for this function:

## Member Function Documentation

|  |  |  |  |  |  |
| --- | --- | --- | --- | --- | --- |
| boolean umms.core.coordinatesystem.TranscriptInGenomicSpace.containsDataFor | ( | String | *chromosome* | ) |  |

Returns true if this coordinate space contains data for the specified chromosome

|  |  |  |  |  |
| --- | --- | --- | --- | --- |
| Collection<String> umms.core.coordinatesystem.TranscriptInGenomicSpace.getChromosomeNames | ( |  | ) |  |

Returns
:   A list of chromosome names contained in the coordinate space

Implements umms.core.coordinatesystem.CoordinateSpace.

Here is the call graph for this function:

|  |  |  |  |  |  |
| --- | --- | --- | --- | --- | --- |
| Annotation umms.core.coordinatesystem.TranscriptInGenomicSpace.getEntireChromosome | ( | String | *chrName* | ) |  |

Get an annotation spanning an entire chromosome

Parameters
:   |  |  |
    | --- | --- |
    | chrName | The chromosome name |

Returns
:   Annotation consisting of the entire chromosome

Implements umms.core.coordinatesystem.CoordinateSpace.

|  |  |  |  |
| --- | --- | --- | --- |
| Collection<? extends Window> umms.core.coordinatesystem.TranscriptInGenomicSpace.getFragment | ( | String | *chr*, |
|  |  | int | *start*, |
|  |  | int | *end* |
|  | ) |  |  |

Returns a fragment of type Window between start and end on chromosome

Parameters
:   |  |  |
    | --- | --- |
    |  |  |

Implements umms.core.coordinatesystem.CoordinateSpace.

Here is the caller graph for this function:

|  |  |  |  |  |  |
| --- | --- | --- | --- | --- | --- |
| Collection<? extends Window> umms.core.coordinatesystem.TranscriptInGenomicSpace.getFragment | ( | Annotation | *annotation* | ) |  |

Returns a fragment of type Window between start and end on chromosome

Parameters
:   |  |  |
    | --- | --- |
    | Annotation |  |

Returns

Implements umms.core.coordinatesystem.CoordinateSpace.

Here is the call graph for this function:

|  |  |  |  |  |
| --- | --- | --- | --- | --- |
| long umms.core.coordinatesystem.TranscriptInGenomicSpace.getLength | ( |  | ) |  |

Returns the total number of positions in the coordinate space For non-contiguous coordinate spaces, this will return a collapsed count such that overlapping blocks are not double counted

Returns
:   number of positions in space

Implements umms.core.coordinatesystem.CoordinateSpace.

Here is the caller graph for this function:

|  |  |  |  |  |  |
| --- | --- | --- | --- | --- | --- |
| long umms.core.coordinatesystem.TranscriptInGenomicSpace.getLength | ( | String | *chr* | ) |  |

Returns the total number of positions in the coordinate space For non-contiguous coordinate spaces, this will return a collapsed count such that overlapping blocks are not double counted

Parameters
:   |  |  |
    | --- | --- |
    | only | positions on a given chromosome |

Returns
:   number of positions in space

Implements umms.core.coordinatesystem.CoordinateSpace.

|  |  |  |  |
| --- | --- | --- | --- |
| Collection<? extends Window> umms.core.coordinatesystem.TranscriptInGenomicSpace.getOverlappingRegion | ( | String | *chr*, |
|  |  | int | *start*, |
|  |  | int | *end* |
|  | ) |  |  |

Returns an interval tree over the genomic region

Implements umms.core.coordinatesystem.CoordinateSpace.

Here is the call graph for this function:

|  |  |  |  |  |  |
| --- | --- | --- | --- | --- | --- |
| Annotation umms.core.coordinatesystem.TranscriptInGenomicSpace.getReferenceAnnotation | ( | String | *name* | ) |  |

Parameters
:   |  |  |
    | --- | --- |
    | name |  |

Returns
:   The named reference as an annotation

Implements umms.core.coordinatesystem.CoordinateSpace.

Here is the call graph for this function:

Here is the caller graph for this function:

|  |  |  |  |  |
| --- | --- | --- | --- | --- |
| final Collection<? extends Annotation> umms.core.coordinatesystem.TranscriptInGenomicSpace.getReferenceAnnotations | ( |  | ) |  |

Returns
:   The reference annotations contained in this coordinate space.

Implements umms.core.coordinatesystem.CoordinateSpace.

Here is the call graph for this function:

|  |  |  |  |  |
| --- | --- | --- | --- | --- |
| final Collection<String> umms.core.coordinatesystem.TranscriptInGenomicSpace.getReferenceNames | ( |  | ) |  |

Returns
:   A list of reference names contained in the coordinate space (e.g. chr1, chr2, etc.)

Implements umms.core.coordinatesystem.CoordinateSpace.

Here is the caller graph for this function:

|  |  |  |  |  |  |
| --- | --- | --- | --- | --- | --- |
| int umms.core.coordinatesystem.TranscriptInGenomicSpace.getSize | ( | Annotation | *region* | ) |  |

Get the size of the annotation with respect to the coordinate space

Parameters
:   |  |  |
    | --- | --- |
    | region |  |

Returns

Implements umms.core.coordinatesystem.CoordinateSpace.

Here is the call graph for this function:

|  |  |  |  |  |  |
| --- | --- | --- | --- | --- | --- |
| long umms.core.coordinatesystem.TranscriptInGenomicSpace.getUnmaskedLength | ( | String | *chr* | ) |  |

Here is the call graph for this function:

|  |  |  |  |
| --- | --- | --- | --- |
| Iterator<Window> umms.core.coordinatesystem.TranscriptInGenomicSpace.getWindowIterator | ( | int | *windowSize*, |
|  |  | String | *chr*, |
|  |  | int | *start*, |
|  |  | int | *end*, |
|  |  | int | *overlap* |
|  | ) |  |  |

Returns an iterator on chromosome chr starting at start, ending at end, of size, windowSize with overlap between consecutive windows

Implements umms.core.coordinatesystem.CoordinateSpace.

Here is the caller graph for this function:

|  |  |  |  |
| --- | --- | --- | --- |
| Iterator<Window> umms.core.coordinatesystem.TranscriptInGenomicSpace.getWindowIterator | ( | int | *windowSize*, |
|  |  | String | *chr*, |
|  |  | int | *start*, |
|  |  | int | *end*, |
|  |  | int | *overlap*, |
|  |  | boolean | *backward* |
|  | ) |  |  |

|  |  |  |  |
| --- | --- | --- | --- |
| Iterator<? extends Window> umms.core.coordinatesystem.TranscriptInGenomicSpace.getWindowIterator | ( | Annotation | *window*, |
|  |  | int | *windowSize*, |
|  |  | int | *overlap* |
|  | ) |  |  |

Returns
:   returns an iterator over an annotation sequentially

Implements umms.core.coordinatesystem.CoordinateSpace.

Here is the call graph for this function:

|  |  |  |  |
| --- | --- | --- | --- |
| Iterator<? extends Window> umms.core.coordinatesystem.TranscriptInGenomicSpace.getWindowIterator | ( | Annotation | *window*, |
|  |  | int | *windowSize*, |
|  |  | int | *overlap*, |
|  |  | boolean | *considerStrand* |
|  | ) |  |  |

Here is the call graph for this function:

|  |  |  |  |
| --- | --- | --- | --- |
| Iterator<Window> umms.core.coordinatesystem.TranscriptInGenomicSpace.getWindowIterator | ( | String | *chr*, |
|  |  | int | *windowSize*, |
|  |  | int | *overlap* |
|  | ) |  |  |

Get a window iterator from start to finish on chromosome chr

Implements umms.core.coordinatesystem.CoordinateSpace.

Here is the call graph for this function:

|  |  |  |  |
| --- | --- | --- | --- |
| Iterator<Window> umms.core.coordinatesystem.TranscriptInGenomicSpace.getWindowIterator | ( | int | *windowSize*, |
|  |  | int | *overlap* |
|  | ) |  |  |

Get a window iterator from start to finish in the entire space

Implements umms.core.coordinatesystem.CoordinateSpace.

|  |  |  |  |
| --- | --- | --- | --- |
| Iterator<Window> umms.core.coordinatesystem.TranscriptInGenomicSpace.getWindowIterator | ( | Collection< Gene > | *baseGenes*, |
|  |  | int | *windowSize*, |
|  |  | int | *overlap* |
|  | ) |  |  |

Get a window iterator over a collection of GeneWindows

Parameters
:   |  |  |
    | --- | --- |
    | baseGenes | The regions over which to iterate |
    | windowSize | Window size |
    | overlap | Overlap size |

Returns
:   Iterator over windows on the collection of regions

Implements umms.core.coordinatesystem.CoordinateSpace.

|  |  |  |  |  |  |
| --- | --- | --- | --- | --- | --- |
| boolean umms.core.coordinatesystem.TranscriptInGenomicSpace.hasChromosome | ( | String | *chr* | ) |  |

Returns true if the coordinate space contains any annotations on the queried chromosome

Parameters
:   |  |  |
    | --- | --- |
    | chr |  |

Returns

Implements umms.core.coordinatesystem.CoordinateSpace.

|  |  |  |  |  |  |
| --- | --- | --- | --- | --- | --- |
| boolean umms.core.coordinatesystem.TranscriptInGenomicSpace.isValidWindow | ( | Annotation | *window* | ) |  |

Check if window in coordinate space and not masked

Parameters
:   |  |  |
    | --- | --- |
    | window |  |

Returns

Implements umms.core.coordinatesystem.CoordinateSpace.

Here is the call graph for this function:

|  |  |  |  |  |  |
| --- | --- | --- | --- | --- | --- |
| Annotation umms.core.coordinatesystem.TranscriptInGenomicSpace.permuteAnnotation | ( | Annotation | *a* | ) |  |

Permutes an annotation to a new location on the same reference in the given coordinate space, preserving the length of the annotation and maintaining a consistent block structure. Does not check whether the given annotation was valid in the coordinate space in the first place.

Parameters
:   |  |  |
    | --- | --- |
    | a | Annotation to permute. Will be modified. |

Returns
:   The permuted Annotation

Exceptions
:   |  |  |
    | --- | --- |
    | PermutationNotFoundException | if an annotation cannot be found |

Implements umms.core.coordinatesystem.CoordinateSpace.

Here is the call graph for this function:

|  |  |  |  |
| --- | --- | --- | --- |
| Annotation umms.core.coordinatesystem.TranscriptInGenomicSpace.permuteAnnotation | ( | Annotation | *a*, |
|  |  | Annotation | *bounds* |
|  | ) |  |  |

Permutes an annotation to a new location within the given bounds, preserving the length of the annotation and maintaining a consistent block structure. Does not check whether the given annotation was valid in the coordinate space in the first place.

Parameters
:   |  |  |
    | --- | --- |
    | a | Annotation to permute. Will be modified. |
    | bounds | Boundaries within which the annotation will be permuted |

Returns
:   The permuted Annotation

Exceptions
:   |  |  |
    | --- | --- |
    | PermutationNotFoundException | if a permuted location cannot be found |

Implements umms.core.coordinatesystem.CoordinateSpace.

Here is the call graph for this function:

|  |  |  |  |  |  |
| --- | --- | --- | --- | --- | --- |
| void umms.core.coordinatesystem.TranscriptInGenomicSpace.setPercentMaskedAllowed | ( | double | *pct* | ) |  |

Here is the caller graph for this function:

|  |  |  |  |  |  |
| --- | --- | --- | --- | --- | --- |
| static void umms.core.coordinatesystem.TranscriptInGenomicSpace.setSeed | ( | long | *seed* | ) |  |

static

Set the seed for the random generator (used for permuting regions).

## Member Data Documentation

final int umms.core.coordinatesystem.TranscriptInGenomicSpace.PERMUTATION\_ATTEMPTS = 10

static

---

The documentation for this class was generated from the following file:

- java/umms/core/coordinatesystem/TranscriptInGenomicSpace.java


---

Generated on Fri Aug 15 2014 15:11:10 for ESAT by  

 1.8.7
